# Isoliquiritigenin Attenuates *Staphylococcus aureus* Adhesion and Invasion to Counteract *Staphylococcus aureus* Pathogenicity and Infection

**DOI:** 10.3389/fcimb.2025.1686699

**Published:** 2025-11-14

**Authors:** Lili Tian, Jian Sun, Hong Jiang, Hongjun Wang, Jian Zheng, Dacheng Wang, Libo Zhang

**Affiliations:** 1 College of Animal Science and Veterinary Medicine (Affiliated Animal Hospital), Jinzhou Medical University, Jinzhou, Liaoning, China; 2 Liaoning Provincial Key Laboratory of Animal Product Quality and Safety, Jinzhou, Liaoning, China; 3 Department of Animal Husbandry and Veterinary Medicine, Beijing Vocational College Agriculture, Beijing, China; 4 College of Animal Science, Jilin University, Changchun, China

**Keywords:** methicillin-resistant staphylococcus aureus, isoliquiritigenin, sortase A, antivirulence therapy, antibiotic resistance, pneumonia

## Abstract

**Introduction:**

Methicillin-resistant *Staphylococcus aureus* (MRSA) is a major clinical challenge due to its virulence and multidrug resistance. Antivirulence strategies targeting key pathogenic mechanisms without affecting bacterial viability provide a promising alternative to conventional antibiotics.

**Methods:**

The inhibitory effect of isoliquiritigenin (ISL) on *S. aureus* sortase A (SrtA) was assessed using a fluorescence resonance energy transfer assay. Fluorescence quenching and molecular docking analyses were performed to elucidate the binding interaction between ISL and SrtA. Adhesion and biofilm formation were evaluated on fibrinogen- and fibronectin-coated surfaces, and bacterial growth was monitored to confirm non-bactericidal activity. The therapeutic efficacy of ISL was further examined in a murine pneumonia model through bacterial load quantification, histopathological analysis, and survival evaluation.

**Results:**

ISL inhibited SrtA activity in a dose-dependent manner (IC_50_ = 13.34 µg/mL), disrupted adhesion and biofilm formation without affecting bacterial growth, and bound reversibly to key catalytic residues of SrtA. In vivo, ISL treatment significantly reduced pulmonary bacterial burden, alleviated tissue damage, and improved survival in infected mice.

**Discussion:**

ISL effectively attenuates MRSA pathogenicity by targeting SrtA-mediated virulence rather than bacterial viability. These results highlight ISL as a promising antivirulence agent and a potential adjuvant for combating antibiotic-resistant S. aureus infections.

## 1 Introduction

The World Health Organization (WHO) has issued a critical warning regarding the rapid and alarming rise of antibiotic-resistant pathogens, which has now escalated to a point of global emergence, requiring immediate, coordinated international action (Alhadrami et al., 2020). In the United States alone, approximately 2.8 million people contract infections caused by antibiotic-resistant bacteria each year, leading to more than 35,000 deaths (Melo et al., 2021). These “superbugs” are proliferating worldwide, driven by novel resistance mechanisms that severely limit our ability to effectively treat both common infectious diseases and hospital-acquired infections. Among the most pressing concerns is methicillin-resistant *Staphylococcus aureus* (MRSA), a pathogen responsible for widespread outbreaks, with mortality rates from invasive infections approaching 20% (Klevens et al., 2007). *Staphylococcus aureus* is a leading pathogen responsible for both hospital-associated and community-acquired infections, contributing to a wide spectrum of human diseases (Tong et al., 2025; Nazli et al., 2024).

Antibiotic resistance represents a formidable and growing threat to global health, as bacterial pathogens have evolved a wide range of mechanisms to evade the effects of traditional antibiotics. These mechanisms include impeding drug entry, modifying antibiotic targets, and developing enzymatic processes that degrade or alter antibiotics (Walsh, 2000). Despite advancements in drug development, current therapies offer limited means of bypassing these resistance pathways, necessitating innovative approaches. One promising strategy is to target bacterial virulence factors rather than focus solely on bacterial growth inhibition or eradication. This approach offers the potential to disarm pathogens without imposing selective pressure for resistance. Among the most promising virulence targets is sortase A (SrtA), a cysteine transpeptidase that plays a key role in *Staphylococcus aureus* pathogenicity (Maresso and Schneewind, 2008).

Sortase A (SrtA) not only plays a critical role in the attachment of surface proteins to the bacterial cell wall but also has profound implications for bacterial adhesion and biofilm formation, both of which are essential for the pathogenicity of *Staphylococcus aureus* (Mazmanian et al., 1999). Through the anchoring of virulence factors such as clumping factors (ClfA and ClfB), fibronectin-binding proteins (FnBPs), and protein A, SrtA facilitates the adhesion of *S. aureus* to host tissues and surfaces, initiating colonization and infection (Mazmanian et al., 2001; Maresso and Schneewind, 2008; Alharthi et al., 2021). This adhesion is the first step in biofilm formation and involves a structured community of bacteria enclosed in a protective matrix that is resistant to both immune responses and antibiotic treatments. Biofilms significantly increase the ability of bacteria to persist on medical devices and within host tissues, leading to chronic infections (Otto, 2013; Schilcher and Horswill, 2020). Inhibiting SrtA disrupts the initial attachment of *S. aureus* to surfaces, thereby preventing the development of biofilms. By targeting the enzyme responsible for this crucial step, SrtA inhibitors not only impair bacterial adhesion but also hinder the establishment and maturation of biofilms. This is especially important because biofilms are notoriously difficult to eradicate and contribute to the persistence of infections, particularly in healthcare settings. Recent studies have shown that natural products with inhibitory effects on SrtA can significantly reduce biofilm formation, suggesting a promising strategy to counteract biofilm-associated infections without directly affecting bacterial growth or viability. This selective approach helps mitigate the risk of antibiotic resistance, making SrtA an important therapeutic target in the fight against bacterial adhesion and biofilm formation (Foster, 2019; Bentley et al., 2008).

Over the past two decades, SrtA has emerged as a focal point in the development of new therapeutic agents aimed at reducing bacterial virulence (Si et al., 2016). Despite its promising potential, challenges persist in the design of effective inhibitors, primarily due to the flexible conformation and structural intricacies of SrtA’s active site, which complicate precise inhibitor binding. Recent efforts have increasingly focused on natural products, given their broad chemical diversity and ability to engage complex biological targets. Natural product-derived inhibitors have shown significant potential in modulating the enzymatic function of SrtA, thus attenuating virulence.

In this study, we employed a high-throughput fluorescence resonance energy transfer (FRET) assay to screen a comprehensive library of natural products, ultimately identifying isoliquiritigenin as a potent inhibitor of SrtA. Isoliquiritigenin, a flavonoid with well-documented anti-inflammatory and antioxidant activities, strongly inhibits the enzymatic function of SrtA. Notably, this is the first study to identify isoliquiritigenin as a direct SrtA inhibitor through high-throughput screening, revealing a previously unrecognized antivirulence mechanism. Mechanistic studies revealed that isoliquiritigenin interacts directly with the active site of SrtA, inhibiting the covalent linkage of surface proteins to the bacterial cell wall. This inhibition not only blocks essential virulence mechanisms but also significantly attenuates *S. aureus* pathogenicity *in vivo*. This approach aligns with the growing paradigm of antivirulence therapy, offering a promising alternative to conventional antibiotics in the ongoing battle against multidrug-resistant pathogens such as MRSA.

## 2 Materials and methods

### 2.1 Bacterial strains, plasmids, and culture conditions

The methicillin-resistant *Staphylococcus aureus* (MRSA) USA300 strain and methicillin-sensitive *S. aureus* (MSSA) Newman strain utilized throughout this study were sourced from the American Type Culture Collection (ATCC; Manassas, VA, United States). The pET28-SrtA expression vector and the *srtA* deletion mutant (Δ*srtA*) were generously provided by Dr. Xuming Deng. SA28 and SA34 are clinical isolates obtained from the Affiliated Hospital of Liaoning University of Traditional Chinese Medicine. The *Escherichia coli* BL21 (DE3) strain served as the bacterial host for expression vector construction. For bacterial culture, *S. aureus* and *E. coli* were grown in brain heart infusion (BHI, Hopebio, Qingdao, China) and Luria–Bertani (LB) broth, respectively, with shaking at 220 rpm at 37 °C. Where necessary, kanamycin (100 µg/mL) was included for plasmid selection or maintenance.

### 2.2 Protein expression and purification

The pET28-SrtA expression vector was transformed into *E. coli* BL21 Gold (DE3) cells (Tiangen, Beijing, China), which were subsequently cultured in LB media supplemented with 50 μg/mL kanamycin. When the optical density (OD_600_) of the culture reached ~0.8, protein expression was induced with 1 mM isopropyl-β-D-thiogalactoside (IPTG) for 16 hours at 20 °C. The harvested cells were resuspended in lysis buffer (20 mM Tris-HCl, pH 7.5, 150 mM NaCl, and 5 mM CaCl_2_) and disrupted by sonication. After centrifugation (30 minutes at 10,000 rpm), the supernatant containing the target protein was applied to a HisTrap HP 5 mL column (GE Healthcare, Chicago, IL, USA) for affinity purification. Further purification was achieved via gel filtration via a HiLoad 16/60 Superdex 200 column (GE Healthcare). The purified SrtA was concentrated, aliquoted, and stored at −80 °C following flash freezing in liquid nitrogen. Protein concentrations were determined by measuring the absorbance at 280 nm, and the purity was verified via SDS–PAGE.

### 2.3 SrtA inhibition assay

The inhibitory activities of the test compounds against *S. aureus* SrtA were evaluated via established methods. In brief, recombinant SrtA (1 µM final concentration) was incubated in assay buffer supplemented with 10 µM Abz-LPETG-Dap (Dnp)-NH_2_. Inhibitors dissolved in DMSO were added to the reaction mixture, and the assay was initiated by the addition of SrtA. The reaction was monitored for 30 minutes at 30 °C using an Infinite M200 Pro plate reader (Tecan, Männedorf, Switzerland) with excitation at 395 nm and emission at 420 nm (Hou et al., 2018).

### 2.4 Determination of IC_50_ values

The IC_50_ values were determined by incubating 1 µM *S. aureus* SrtA in reaction buffer with serial dilutions of each test compound (ranging from 0 to 64 µg/mL) at room temperature for 20 minutes. IC_50_ values were calculated via GraphPad Prism software, which employs sigmoidal dose–response curve fitting.

### 2.5 Irreversible inhibition assay

Purified SrtA (5 µM) was incubated with isoliquiritigenin at its IC_50_ or with DMSO as a control at room temperature for 1 hour. Following incubation, the reaction mixture was subjected to centrifugal filtration to remove any unbound inhibitor. Protein aliquots (25 µL) collected before and after filtration were added to 10 µM Abz-LPATG-Dnp substrate. The fluorescence intensity was measured every minute for 20 minutes via a FlexStation 3 (Molecular Devices), with the excitation and emission wavelengths set at 309 nm and 420 nm, respectively (Zhang et al., 2014).

### 2.6 Bacterial growth curve assay

Overnight cultures of *S. aureus* were diluted 1:1000 in fresh tryptic soy broth (TSB) medium and cultured with various concentrations of ISL. The A_600_ values were measured every hour over an 18-hour period via a microplate reader. Growth curves were generated and analyzed via GraphPad Prism software. Each experiment was conducted in triplicate to ensure reproducibility.

### 2.7 Minimum inhibitory concentration assay

The minimum inhibitory concentrations (MICs) of isoliquiritigenin were determined for *S. aureus* USA300 or Newman. Overnight cultures were grown in TSB, diluted 1:100, and incubated at 37 °C for 2–3 hours until the optical density at 600 nm (A_600_) reached approximately 0.6. The cultures were then further diluted 1:400, and 200 µL of the resulting bacterial suspension was added to each well of a 96-well microtiter plate containing twofold serial dilutions of the compounds. The plates were incubated at 37 °C for 16–18 hours, after which A600 values were measured to assess bacterial growth. MIC values were defined as the lowest concentration of the compounds that completely inhibited visible growth, as indicated by the A_600_ readings (Andrews, 2001).

### 2.8 Erythrocyte stability assay

The erythrocyte suspension was incubated with increasing concentrations of isoliquiritigenin (0–128 µg/mL) at 37 °C for 30 minutes. Triton X-100 (0.1%) was used as a positive control for hemolysis, while PBS-treated erythrocytes served as the negative control. Following incubation, the samples were centrifuged at 3000 rpm for 10 minutes to pellet the erythrocytes. The supernatant was carefully collected, and the extent of hemolysis was assessed visually by measuring the absorbance of the supernatant at 543 nm via a microplate reader to quantify the released hemoglobin.

### 2.9 MTT assay for assessing the cytotoxicity of isoliquiritigenin in A549 and 293T cells

A549 (human lung carcinoma) and 293T (human embryonic kidney) cells were cultured in Dulbecco’s modified Eagle’s medium (DMEM; Gibco, USA) supplemented with 10% fetal bovine serum (FBS; Gibco, USA), 100 U/mL penicillin, and 100 µg/mL streptomycin in a humidified incubator at 37 °C with 5% CO_2_. The cytotoxicity of isoliquiritigenin was evaluated via the MTT assay. The cells were seeded into 96-well plates at a density of 5 × 10^3^ cells per well in 100 µL of complete DMEM and allowed to adhere overnight. After 24 hours, the cells were treated with various concentrations of isoliquiritigenin (ranging from 0–32 μg/mL) prepared in DMEM and incubated for an additional 24 hours. Following the treatment period, 10 µL of 5 mg/mL MTT solution (Beyotime, Beijing, China) was added to each well and incubated at 37 °C for 4 hours. Afterward, the medium was carefully removed, and the resulting formazan crystals were dissolved in 100 µL of dimethyl sulfoxide (DMSO). The absorbance was measured at 570 nm via a microplate reader.

### 2.10 Drug safety assessment in the *Galleria mellonella* model

*G. mellonella* larvae, measuring 2–2.5 cm in length with a creamy appearance, were selected for the safety assessment. Prior to the experiment, the larvae were kept at room temperature and fasted for 24 hours. The study included four groups: a control group, a solvent control group (5% DMSO, 20% PEG300, and 75% PBS), and two treatment groups receiving isoliquiritigenin at concentrations of 20 mg/kg and 40 mg/kg. To administer the compounds, 10 μL of the appropriate solution was injected into the final proleg of each larva via a Hamilton syringe needle. Following injection, the larvae were housed in Petri dishes and incubated at 37 °C. Survival was tracked over a 120-hour period, with observations recorded every 24 hours. Signs of toxicity, such as melanization or lack of movement, were documented through photographs taken 24 hours postinjection (Li et al., 2020).

### 2.11 Biofilm formation

Overnight cultures of *S. aureus* USA300 and Newman were diluted 1:1000 with fresh TSB medium. Two hundred microliter aliquots were added to the wells of 96-well microtiter plates with various concentrations of ISL (0–32 μg/mL) and cultured without shaking at 37 °C for 18 h. The medium was then discarded, and the wells were gently rinsed two times with PBS. The biofilm was immobilized with 200 μL of methanol for 10 min and subsequently stained with 0.1% (w/v) crystal violet solution for 15 min. Excess stain was discarded, and the plates were washed three times with sterile distilled water before being photographed. For quantification, the absorbance of the crystal violet stain dissolved in 33% (v/v) acetic acid solution was measured at 600 nm via a microplate reader (Wang et al., 2021).

### 2.12 Impact of isoliquiritigenin on mature biofilms

To evaluate the effects of isoliquiritigenin on preformed biofilms, *S. aureus* USA300 and Newman strains were cultured in TSB for 24 hours under static conditions at 37 °C to allow mature biofilm development. After 24 hours, the medium was replaced with fresh TSB containing different concentrations of isoliquiritigenin (0–32 µg/mL), and the plates were further incubated for an additional 18 hours. Following treatment, biofilms were processed as described above, including washing with PBS, fixation with methanol, crystal violet staining, and absorbance measurement at 600 nm. This approach allowed the assessment of the capacity of isoliquiritigenin to disrupt or inhibit mature biofilm structures.

### 2.13 FITC-IgG binding assay

Overnight cultures of *S. aureus* were diluted 1:1000 in fresh TSB medium and treated with various concentrations of the inhibitors. The cultures were grown until the optical density at 600 nm (A_600_) reached 1.0. A 600 µL aliquot from each culture was collected via centrifugation at 12000 rpm for 5 minutes. The resulting cell pellets were washed three times with PBS and resuspended in 400 µL of PBS containing 4 µL of FITC-labeled human IgG at a concentration of 0.5 mg/mL. The samples were incubated in the dark at room temperature with gentle shaking for 30 minutes. Following incubation, the cells were pelleted by centrifugation and washed three times with PBS to remove unbound IgG. The fluorescence intensity of FITC was then measured via flow cytometry to evaluate the levels of protein A (SpA) on the bacterial surface (Wang et al., 2021).

### 2.14 Bacterial adherence assay

*S. aureus* strains USA300, SA28, and SA34 were cultured overnight and subsequently diluted 1:100 in fresh BHI medium supplemented with or without isoliquiritigenin. The bacterial suspensions were then incubated at 37 °C with shaking at 180 rpm until the optical density at 600 nm (A600)2 reached 0.5. Next, 100 µL of the bacterial culture was transferred into a polystyrene Costar 96-well plate precoated with 20 µg/mL bovine fibrinogen and stored at 4 °C overnight. After the plate was incubated at 37 °C for 2 hours, the bacterial suspension was carefully removed, and the wells were washed twice with PBS to eliminate nonadherent cells. Adherent bacteria were fixed by treating the wells with 25% formaldehyde for 30 minutes. The wells were then washed twice with PBS and stained with crystal violet for 20 minutes. The absorbance at 570 nm was subsequently measured via a microplate reader. As a positive control, the Δ*srtA* strain underwent identical treatments under the same experimental conditions.

### 2.15 Western blot analysis

Overnight cultures of *S. aureus* were diluted 1:1000 in fresh TSB medium and treated with inhibitors at various concentrations, and growth continued until the A_600_ reached 2.0. A 1 mL aliquot of each culture was collected by centrifugation at 12,000 rpm for 5 minutes, and the resulting cell pellet was washed three times with PBS. The cells were resuspended in 500 µL of PBS containing 10 µg/mL lysostaphin and incubated at 37 °C for 15 minutes. After incubation, the samples were centrifuged at 12,000 rpm for 30 minutes at 4 °C to precipitate the protoplasts. The supernatant, containing cell wall-associated proteins, was collected and mixed with 5× SDS loading buffer. Proteins were resolved by SDS–PAGE and transferred onto nitrocellulose membranes (Millipore, USA). The membranes were blocked with 5% skim milk for 1 hour at room temperature and then incubated overnight at 4 °C with antibodies against SrtA. After incubation with horseradish peroxidase-conjugated goat anti-rabbit IgG as the secondary antibody, the immunoreactive bands were visualized via a chemiluminescence substrate (Biosharp, China). Images were captured via an enhanced chemiluminescence detection system.

### 2.16 Fluorescence quenching

The purified SrtA protein was diluted to a working concentration of 500 ng/mL in phosphate-buffered saline (PBS). A volume of 100 μL of the diluted protein mixture was added to each well of a flat-bottom 96-well polystyrene microtiter plate, followed by the addition of various concentrations of isoliquiritigenin to assess its binding affinity. The fluorescence spectra of the solutions were measured via a fluorescence spectrophotometer. The excitation wavelength was set at 280 nm, corresponding to the absorption peak of the protein’s aromatic residues (primarily tryptophan). Fluorescence emission spectra were recorded within the 300–400 nm range, capturing potential shifts in fluorescence intensity as a result of protein–ligand interactions. The bandpass for both excitation and emission was fixed at 5 nm to ensure the optimal resolution of the spectral data. The binding affinity of isoliquiritigenin for SrtA was quantified by calculating the association constant (*K_A_*), following a well-established method from prior research.

### 2.17 Molecular docking and dynamics simulation

The methodological details were refined according to established docking practices, as recommended by previous studies (Dalal et al., 2021a; Kumari and Dalal, 2022). The three-dimensional structure of *S. aureus* SrtA was obtained from the Protein Data Bank (PDB ID: 1T2P) and derived from X-ray crystallography data. The 3D structure of the ligand isoliquiritigenin was generated via the ChemBio3D Ultra 12.0 software suite. Docking between SrtA and isoliquiritigenin was performed via AutoDockTools version 1.5.6, following standard molecular docking protocols to identify optimal binding conformations.

Prior to docking, Kollman charges were added to the protein, and Gasteiger charges were assigned to the ligand to ensure proper electrostatic representation. Polar hydrogens were added, and nonpolar hydrogens were merged to prepare the receptor. A grid box was generated to encompass the active site, with a size of 60 × 60 × 60 Å and a grid spacing of 0.375 Å. The grid center was defined around the catalytic cysteine (Cys184) and adjacent residues (His120 and Arg197), which form the active pocket of SrtA.

Molecular dynamics (MD) simulations were then conducted to evaluate the stability of the protein–ligand complexes via previously established methodologies. The simulations were prepared with the AMBER99SB-ILDN force field for the protein and the general Amber force field (GAFF) for the ligand. The systems were solvated in a TIP3P water box, followed by energy minimization and equilibration to ensure system stability. A 100 ns production run was executed on an NVIDIA^®^ Tesla K20c GPU.

Throughout the MD simulations, the root mean square deviation (RMSD), root mean square fluctuation (RMSF), and radius of gyration (Rg) were monitored to assess the stability of the complexes. Hydrogen bond interactions were also tracked over the course of the simulation to gain insights into the protein–ligand interactions. The binding free energy (Δ*G*_bind) was calculated via the molecular mechanics/generalized.

### 2.18 Therapeutic efficacy against MRSA infections in the *G. mellonella* model

The therapeutic efficacy of the tested compounds against *S. aureus* USA300 was evaluated using *G. mellonella* larvae as an infection model. Overnight bacterial cultures were diluted 1:1000 in fresh TSB and incubated at 37 °C until the optical density at 600 nm reached 1.0. The cultures were subsequently centrifuged at 3000 × g, washed, and resuspended in PBS to obtain the desired concentration. For the infection model, 10 μL of the bacterial suspension, containing approximately 2 × 10^6^ CFU per larva of the *S. aureus* USA300 strain, was injected into the larvae. The *S. aureus* Δ*srtA* strain was used as a positive control. The blank control group received a solution comprising 5% DMSO, 20% PEG300, and 75% PBS. One hour postinfection, the test compound isoliquiritigenin was administered at doses of 10 mg/kg and 20 mg/kg. The larvae were incubated at 37 °C, and survival rates were monitored and recorded over a 120-hour period. At the end of the experiment, photographs were taken to evaluate signs of infection, such as melanization. Larval mortality was confirmed by the absence of movement upon physical stimulation. Additionally, to determine the bacterial load, a separate group of larvae (6 per group) was injected with 1 × 10^7^ CFU of *S. aureus* USA300 per larva. At the designated time points, larvae were surface sterilized and homogenized under sterile conditions, and the homogenates were plated for colony-forming unit (CFU) enumeration.

### 2.19 Mouse pneumonia model

C57BL/6J mice (6–8 weeks old, male and female) were obtained from Liaoning Changsheng Biotechnology Co., Ltd. (China) and housed in a standard laboratory environment with free access to food and water. Overnight cultures of *S. aureus* USA300 were diluted 1:100 in TSB medium and grown to an optical density at 600 nm (OD_600_) of 1.0. The bacteria were collected by centrifugation, washed three times with PBS, and resuspended in PBS. The mice were randomly divided into five groups: (i) the blank control group, (ii) the *S. aureus* USA300 infection group, (iii) the ΔsrtA strain infection group, and two treatment groups receiving isoliquiritigenin at doses of 20 mg/kg and 40 mg/kg, which were administered subcutaneously. Each mouse in the infection groups received 30 μL of bacterial suspension containing 2 × 10^8^ CFUs via nasal inhalation. One hour postinfection, treatment was initiated with the subcutaneous administration of isoliquiritigenin, followed by subsequent doses every 12 hours. Survival rates were monitored and recorded every 12 hours for a total of 96 hours. For histopathological and bacterial load assessments, a separate cohort of mice was infected with 30 μL of *S. aureus* suspension (1 × 10^8^ CFUs) via nasal inhalation. The same group allocation and treatment protocols were applied as in the survival study. At 24 hours post infection, the mice were sacrificed by cervical dislocation. The left lungs were excised and subjected to histological examination via hematoxylin and eosin (H&E) staining to evaluate tissue pathology. The right lungs were aseptically removed, weighed, homogenized, and plated on TSB agar for bacterial load quantification. Additionally, the levels of the inflammatory cytokines IFN-γ, IL-6, and TNF-α in the lung perfusates were measured via ELISA kits (cat# M6140, M6149, M6152, US EVERBRIGHT, China) according to the manufacturer’s instructions.

### 2.20 Statistical analysis

All the statistical analyses were conducted via SPSS software version 13.0 (SPSS Inc., Chicago, IL, USA). For comparisons between two groups, Student’s t test was used for normally distributed data, whereas the Mann–Whitney U test was used for nonnormally distributed data. When three or more groups were compared, one-way ANOVA followed by Tukey’s HSD test or Dunnett’s t test was used for normally distributed continuous variables. For nonnormally distributed variables, the Kruskal–Wallis H test was performed, followed by *post hoc* analysis via the Nemenyi test or the Mann–Whitney U test for pairwise comparisons. The data are presented as the means ± standard deviations (SDs) throughout the manuscript.

## 3 Results

### 3.1 ISL as a reversible inhibitor of *S. aureus* sortase A

**Figure 1:**
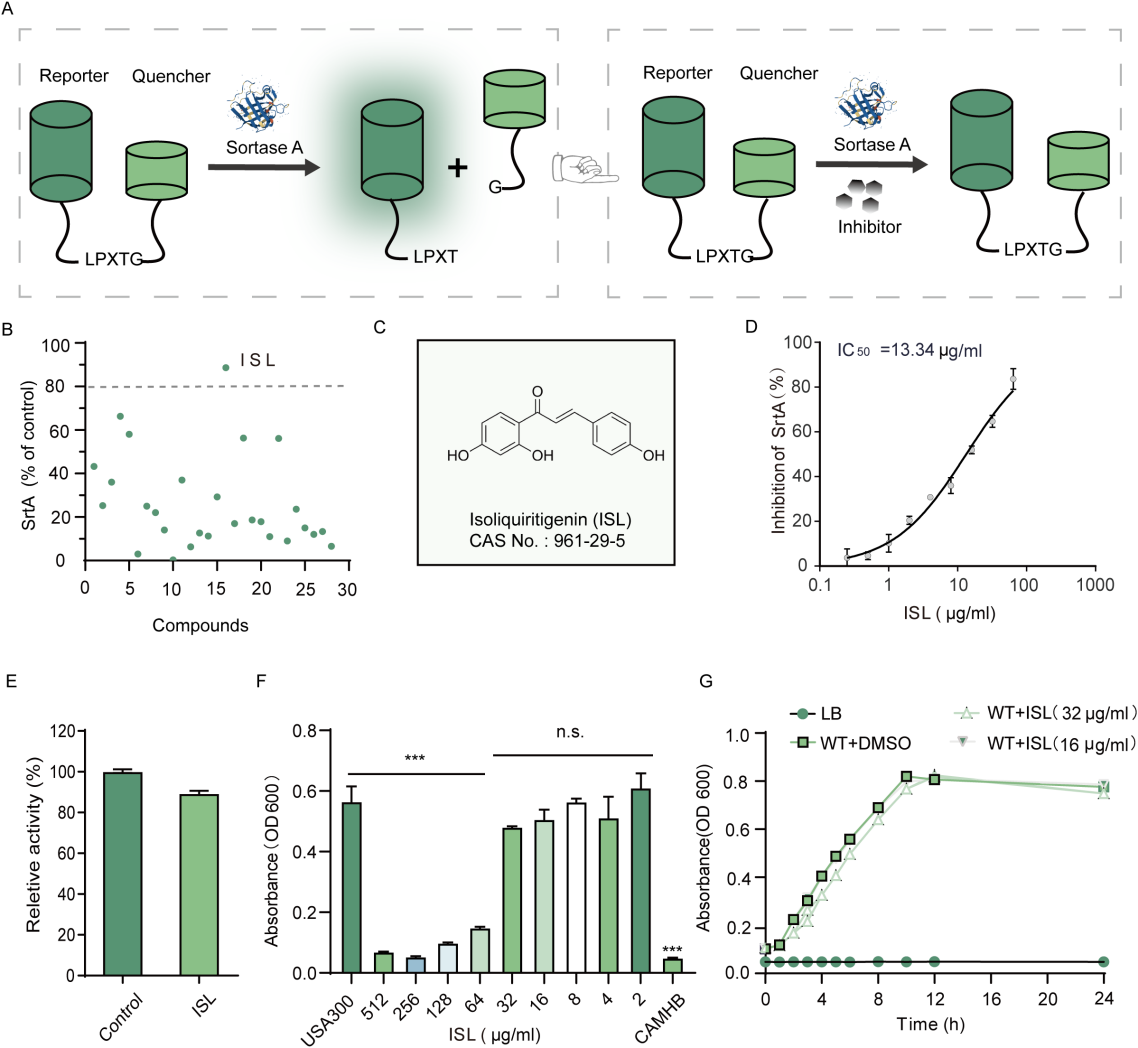
Identification of ISL as an Inhibitor of *Staphylococcus aureus* Sortase A (SrtA). **(A)** FRET-based screening assay used to identify SrtA inhibitors on the basis of their ability to recognize and cleave substrate peptides. **(B)** Among the 30 compounds screened, ISL demonstrated the most potent inhibition of SrtA, resulting in more than 80% inhibition. **(C)** Chemical structure of ISL. **(D)** ISL inhibits SrtA with an IC_50_ value of 13.34 μg/mL, as determined by FRET-based assays. **(E)** Kinetic analysis showing that ISL inhibits SrtA in a reversible, noncovalent manner. **(F)** The minimum inhibitory concentration (MIC) of ISL against the *S. aureus* strain USA300 was 64 μg/mL. **(G)** Growth curves of *S. aureus* strains incubated with sub-MIC concentrations (16 and 32 μg/mL) of ISL showing no significant effect on bacterial growth. “n.s.” denotes not significant (P ≥ 0.05); ***, P < 0.001.

ISL was identified as a potent inhibitor of *S. aureus* sortase A (SrtA) through a FRET-based enzymatic assay (**Figure 1A**). Among a screening panel of 30 natural products, isoliquiritigenin (ISL) demonstrated the highest inhibitory activity, achieving over 80% inhibition, suggesting its selection as a candidate compound for further investigation (**Figures 1B, C**). Subsequent FRET assays confirmed that ISL robustly inhibited SrtA in a dose-dependent manner, with an IC_50_ value of 13.34 μg/mL, indicating its potent inhibitory potential (**Figure 1D**). Kinetic analysis revealed that ISL inhibits SrtA in a reversible and noncovalent manner. This finding was further validated by incubating purified recombinant SrtAΔ_N59_ with ISL at 10× IC_50_ for 1 h, followed by dilution, which resulted in the recovery of SrtA activity to 88.80 ± 1.86% of that of the untreated control (**Figure 1E**). This recovery suggests that ISL does not irreversibly modify the enzyme’s active site, distinguishing it from covalent inhibitors. ISL suppressed Newman growth in a concentration-dependent manner. Complete inhibition (OD_600_ indistinguishable from the medium blank) occurred at 128 µg/mL and above, whereas ≤ 64 µg mL^-^¹ failed to produce a significant reduction in turbidity. Hence, the MIC of ISL against *S. aureus* Newman under the present conditions was 128 µg/mL (**Figure 1F**, **Supplementary Figure 1**). Notably, when *S. aureus* was cultured in the presence of sub-MIC concentrations of ISL (16 and 32 μg/mL), no significant effect on bacterial growth was observed (**Figure 1G**). This finding suggests that while ISL effectively inhibits SrtA activity, it does not exert direct bactericidal effects at concentrations above its IC_50_, which is in line with the antivirulence strategy of targeting bacterial pathogenicity without compromising bacterial viability. These results highlight the potential of ISL as a reversible inhibitor of *S. aureus* SrtA, suggesting a novel approach for modulating bacterial virulence without directly eliminating the pathogen. This mechanism aligns with the therapeutic strategy of selectively targeting bacterial virulence factors, which could reduce the risk of resistance development while preserving the host microbiota.

### 3.2 Safety profile of the ISL

**Figure 2:**
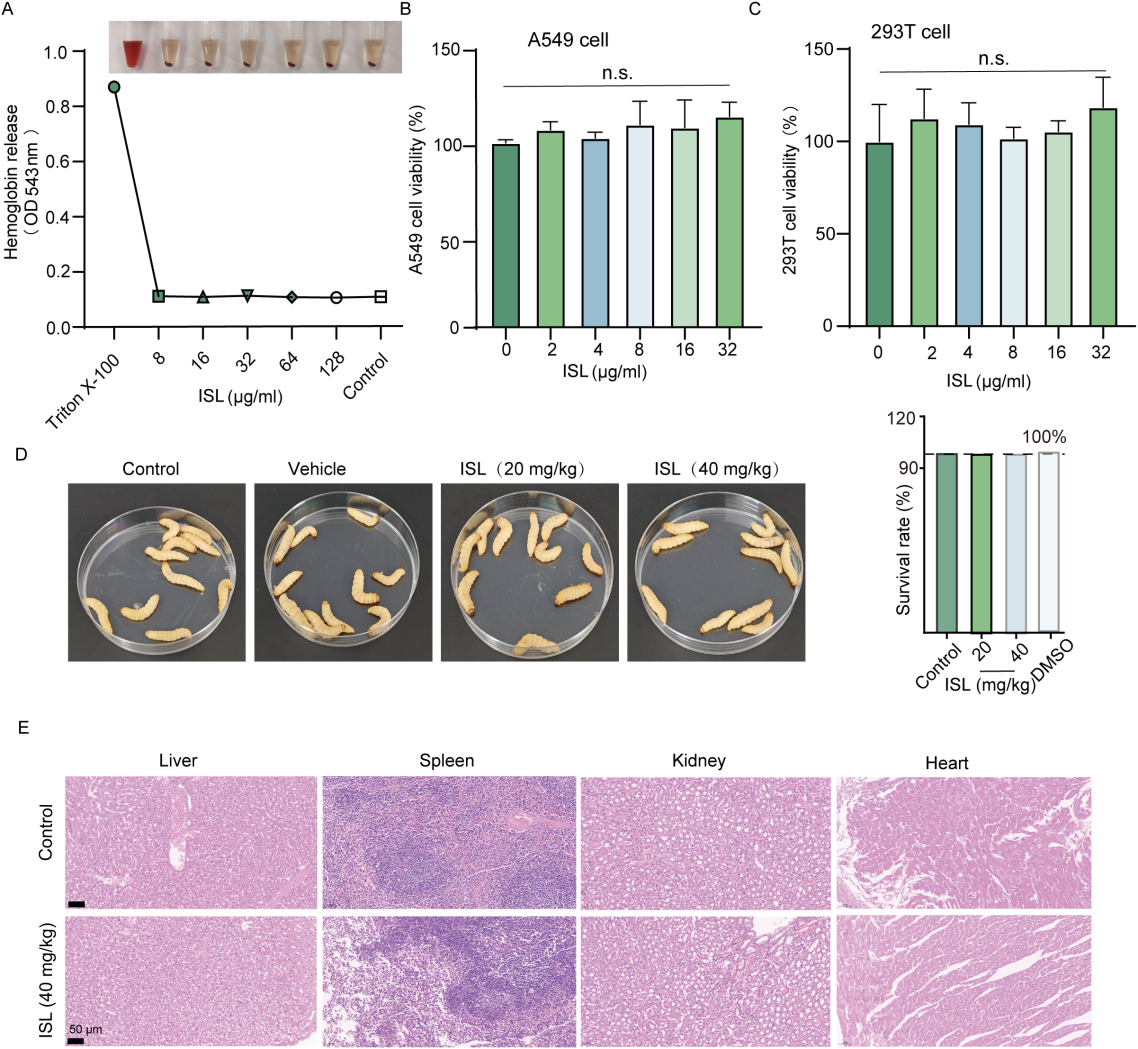
Safety profile of the ISL. **(A)** Hemolytic activity of ISL was assessed using rabbit erythrocytes, revealing no hemolysis at concentrations up to 128 μg/mL. **(B, C)** Cytotoxicity of ISL was evaluated in mammalian A549 and 293T cell lines through the MTT assay. No significant reduction in cell viability was observed, even at concentrations corresponding to the IC_50_ of ISL, indicating minimal cytotoxicity. **(D)***In vivo* safety was assessed in *Galleria mellonella* larvae treated with varying concentrations of ISL. No signs of abnormalities or melanization were observed, even at a dose of 40 mg/kg, with all larvae surviving for 120 hours. **(E)** Histopathological examination of various organ tissues from mice administered ISL (40 mg/kg) revealed no significant differences from those of the control group, further confirming the biocompatibility of ISL. “n.s.” denotes not significant (P ≥ 0.05).

Safety evaluations of ISL revealed no hemolytic activity in rabbit erythrocytes up to concentrations of 128 μg/mL, suggesting a robust safety margin for its potential therapeutic applications (**Figure 2A**). In addition, cytotoxicity was assessed via the MTT assay in mammalian A549 and 293T cell lines. No significant reduction in cell viability was observed at concentrations corresponding to the IC_50_ of ISL, with cell survival rates remaining comparable to those of untreated controls, further confirming its low cytotoxicity (**Figures 2B, C**). To evaluate the *in vivo* safety of ISL, we administered varying concentrations of ISL to *Galleria mellonella* larvae. No abnormalities or melanization were observed, even at a dose of 20 mg/kg, and the larvae exhibited a 100% survival rate over a 120-hour period (**Figure 2D**). Acute toxicity testing in mice also revealed no mortality or abnormal behaviors within 72 h post administration. Histopathological analysis of various organ tissues revealed no significant differences compared with those of the control group (**Figure 2E**). Together, these findings underscore the favorable safety profile of ISL, demonstrating its low cytotoxicity and excellent biocompatibility. These results suggest that ISL holds promise as a therapeutic agent with a low risk of adverse effects, making it a viable candidate for further preclinical and clinical development.

### 3.3 Inhibition of *S. aureus* adhesion, invasion, and biofilm formation by ISL through SrtA suppression

**Figure 3:**
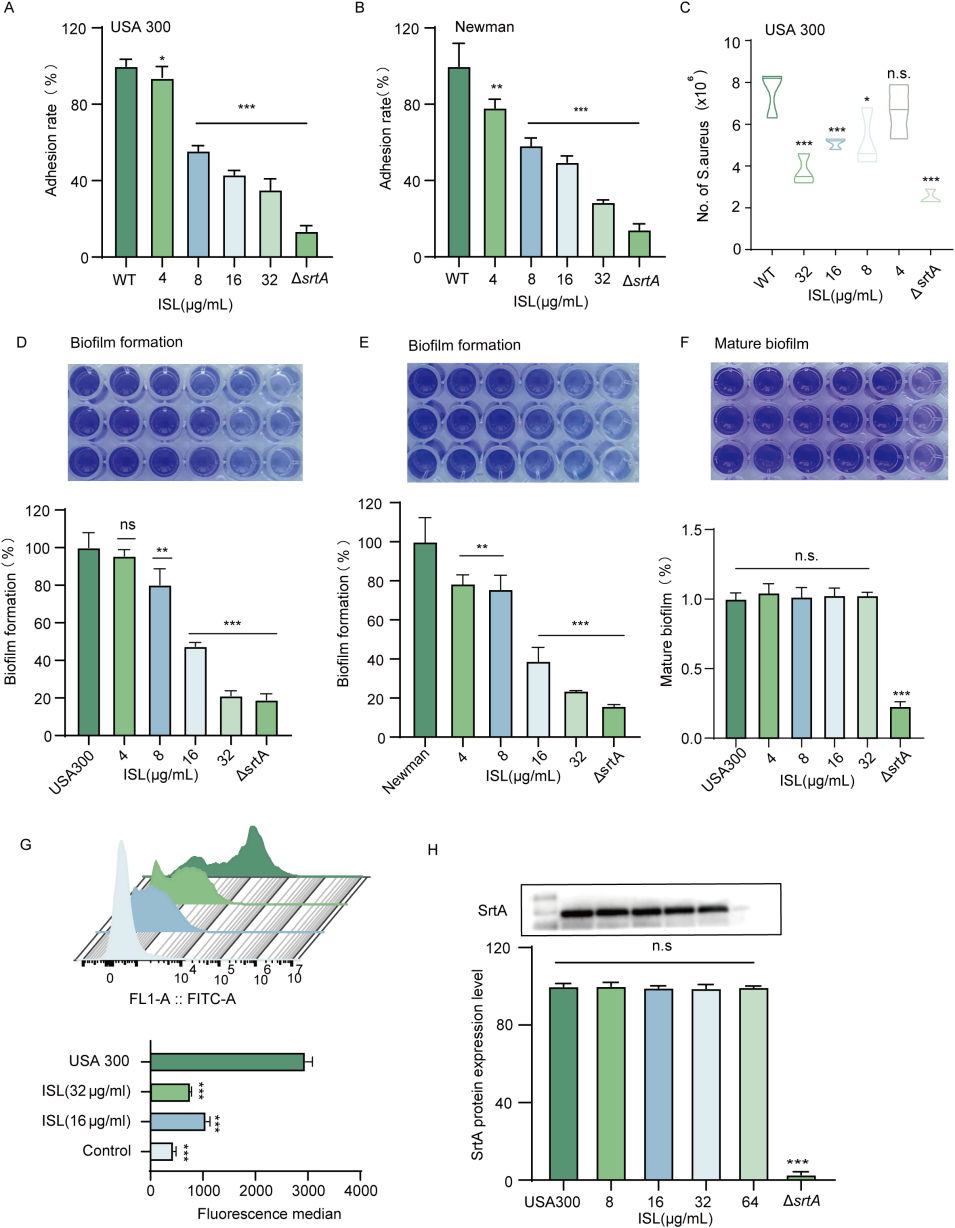
Inhibition of *S. aureus* adhesion, invasion, and biofilm formation by ISL through SrtA suppression. **(A, B)** ISL inhibits *S. aureus* adhesion to fibrinogen in a dose-dependent manner. Treatment with 32 μg/ml ISL reduced the adhesion of *S. aureus* USA300 and Newman. **(C)** ISL significantly reduces the invasive capacity of *S. aureus* USA300 against A549 lung epithelial cells in a concentration-dependent manner, with 32 μg/ml ISL resulting in a marked reduction in bacterial invasion. **(D, E)** ISL inhibits biofilm formation in both *S. aureus* USA300 and Newman strains. **(F)** ISL primarily disrupts the early stages of biofilm formation, with no significant effect on mature biofilms. **(G)** ISL reduces the surface expression of SpA, as evidenced by flow cytometry analysis, indicating that SpA anchoring is suppressed through the inhibition of SrtA activity. **(H)** Western blot analysis revealed that ISL does not alter SrtA expression. n.s., not significant (P ≥ 0.05); *, P < 0.05; **, P < 0.01; ***, P < 0.001.

The absence of the *srtA* gene in *S. aureus* results in a lack of expression of key adhesins, including clumping factors (ClfA, ClfB) and fibronectin-binding proteins (FnbA, FnbB), thereby attenuating bacterial virulence (Oh et al., 2006). Consequently, it is anticipated that inhibitors of *SrtA* could reduce *S. aureus* adhesion to fibrinogen. As shown in **Figure 3A**, increasing concentrations of ISL (4–32 μg/ml) progressively inhibited the adhesion of *S. aureus* USA300 to fibrinogen. Compared with no treatment, treatment with 32 μg/ml ISL reduced the adhesion of *S. aureus* USA300 to fibrinogen. Similar results were observed in the *S. aureus* Newman strain, where treatment with 32 μg/ml ISL reduced fibrinogen adhesion to 28.63± 0.73% (**Figure 3B**). Further investigations were conducted to assess whether ISL affects the invasive capacity of *S. aureus* USA300 against A549 lung epithelial cells. As shown in **Figure 3C**, the number of bacterial colonies within A549 cells significantly decreased in a concentration-dependent manner as the ISL concentration increased. Compared with the control treatment, treatment with 32 μg/ml ISL markedly reduced the number of bacteria invading A549 cells.

The impact of ISL on biofilm formation by *S. aureus* was also evaluated via a static biofilm assay, with biofilm biomass assessed via crystal violet staining and absorbance measurements. As depicted in **Figure 3D**, ISL treatment resulted in a dose-dependent reduction in biofilm formation. At a concentration of 32 µg/mL, ISL markedly inhibited biofilm formation by *S. aureus*, reducing the biomass of the USA300 and Newman strains to 21.25 ± 1.495% and 23.77 ± 0.069% of that of the untreated control, respectively. (**Figure 3E**). Biofilm formation occurs in three stages: initial adhesion and aggregation, biofilm development, and biofilm dispersal. To further investigate this mechanism, we examined the effect of ISL on mature biofilms of *S. aureus* USA300. As shown in **Figure 3F**, treatment with varying concentrations of ISL did not significantly affect mature biofilms. These findings indicate that ISL interferes primarily with the early stages of biofilm formation.

To further assess the impact of ISL on *S. aureus* surface proteins, we examined the surface expression of SpA (protein A), a key surface protein of *S. aureus* USA300, via flow cytometry. Following coincubation with FITC-labeled IgG, which specifically binds to SpA, untreated *S. aureus* exhibited strong fluorescence, indicating robust SpA expression on the bacterial surface. In contrast, treatment with 16 or 32 μg/ml ISL significantly reduced the fluorescence intensity, suggesting that ISL disrupts the anchoring of SpA on the bacterial surface by inhibiting SrtA activity (**Figure 3G**). To further investigate whether ISL affects SrtA protein expression, we performed Western blot analysis. The results demonstrated that ISL treatment did not alter SrtA expression levels, indicating that ISL does not interfere with the transcription or translation of SrtA but rather suppresses its enzymatic activity to inhibit virulence factor anchoring (**Figure 3H**). Together, these findings suggest that ISL inhibits *S. aureus* adhesion, invasion, and biofilm formation primarily through the suppression of SrtA activity.

### 3.4 Direct interaction between ISL and SrtA

**Figure 4:**
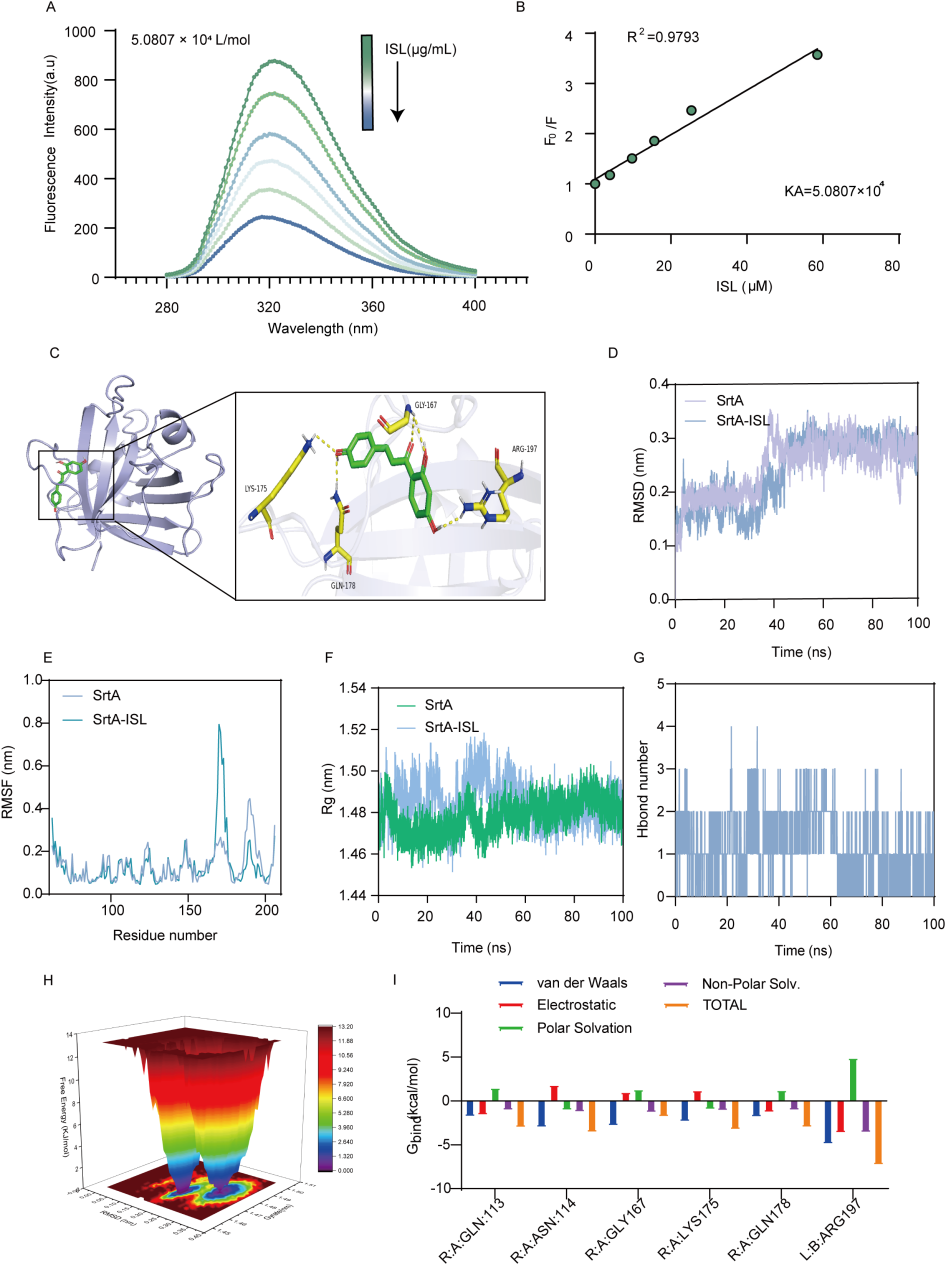
Direct interaction between ISL and SrtA. **(A, B)** Fluorescence quenching assays revealed that increasing concentrations of ISL progressively quenched the fluorescence of SrtA in a concentration-dependent manner, with a strong linear correlation (R² = 0.9793), indicating a high affinity between ISL and SrtA. The calculated binding constant (K_a_ = 5.0807 × 10^4^ L/mol) further supports this interaction. **(C)** Molecular docking studies revealed that ISL interacts with key residues in the catalytic pocket of SrtA, forming hydrogen bonds with LYS-175, GLY-167, GLN-178, and ARG-197, with a docking score of -7.9 kcal/mol. **(D, E)** RMSD and RMSF analyses over a 50 ns simulation demonstrated the stability of the ISL-SrtA complex, with minimal flexibility (RMSF < 0.8 Å) in the binding region, confirming stable interactions. **(F)** Radius of gyration (R_g) analysis revealed no significant conformational changes in SrtA upon ISL binding, further supporting complex stability. **(G)** Hydrogen bond analysis revealed the sustained formation of 2–3 hydrogen bonds between ISL and SrtA. **(H)** Free energy landscape (FEL) analysis confirmed stable binding, with minimal energy conformations overlapping with the average structure. **(I)** MMGBSA analysis reveals the energetic contributions of individual amino acids to the SrtA-ISL interaction.

SrtA contains aromatic amino acids that can emit fluorescence under specific excitation wavelengths, which makes fluorescence quenching assays valuable tools for studying the interaction between ISL and SrtA. As shown in **Figure 4A**, increasing concentrations of ISL led to progressive quenching of SrtA fluorescence, with a strong linear relationship between the ISL concentration and fluorescence intensity (R² = 0.9793; **Figure 4B**). The calculated binding constant (K_a_) of 5.0807 × 10^4^ L/mol further demonstrates the strong affinity of ISL for SrtA. To gain deeper insight into the molecular mechanism of the inhibition of SrtA by ISL, we conducted molecular docking studies. These results revealed that ISL interacts with key residues in the catalytic pocket of SrtA, forming hydrogen bonds with LYS-175, GLY-167, GLN-178, and ARG-197. The docking score of -7.9 kcal/mol suggests a high affinity between ISL and SrtA (**Figure 4C**). We then performed root mean square deviation (RMSD) analysis of the system over a 50 ns simulation to assess the stability of the ISL-SrtA complex. Both the free SrtA protein and the SrtA-ISL complex reached equilibrium within 50 ns (**Figure 4D**), confirming the stability of the binding interaction. Root mean square fluctuation (RMSF) analysis revealed that the residues involved in ISL binding exhibited minimal flexibility (RMSF < 0.8 Å), indicating a rigid and stable binding interface (**Figure 4E**). The radius of gyration (R_g) analysis revealed no significant conformational changes in SrtA upon ISL binding, supporting the stability of the complex (**Figure 4F**). Further solvent-accessible surface area (SASA) analysis revealed a rapid increase in surface area during the first 40 ns, followed by stabilization between 40 and 100 ns, confirming the formation of a stable ISL-SrtA complex (**Supplementary Figure 2**). Hydrogen bond analysis indicated the sustained formation of 2–3 hydrogen bonds between ISL and SrtA, which are essential for maintaining the stability and enhancing the binding strength of the complex (**Figure 4G**). Free energy landscape (FEL) analysis corroborated these findings, revealing significant overlap between the minimal energy and average conformations, which was consistent with the RMSD and R_g results (**Figure 4H**). MMGBSA calculations further revealed that residues, particularly ARG-197, contribute significantly to van der Waals interactions with ISL (**Figure 4I**). The importance of ARG-197 in the catalytic function of SrtA has been well established, and its inhibition leads to enzyme inactivation (Bentley et al., 2008). Additionally, the binding of ISL to SrtA induced notable changes in the protein’s secondary structure, with transitions observed between helices, turns, and loops, reflecting a conformational shift upon ISL binding (**Supplementary Figure 3**). In summary, our molecular docking and dynamic simulations provide compelling evidence that ISL directly interacts with SrtA.

### 3.5 ISL protects *Galleria mellonella* from *S. aureus* infection and reduces melanin production

**Figure 5:**
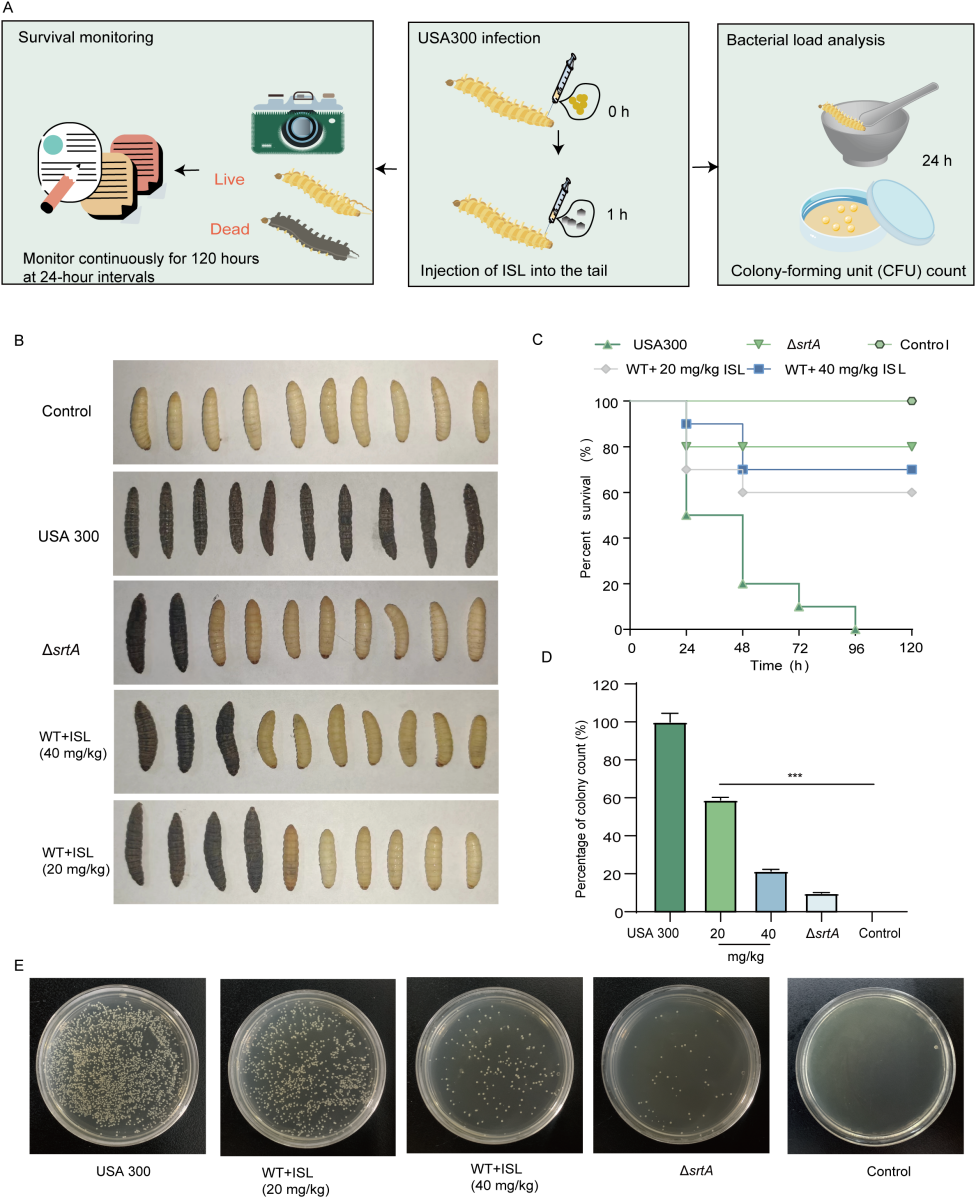
ISL protects *G*. *mellonella* from *S. aureus* infection and reduces melanin production. **(A)** The survival and bacterial load of *G*. *mellonella* larvae were assessed 24 or 120 h post infection with *S. aureus* USA300 and subsequent treatment with ISL. **(B)** At 120 hours, the survival of *G*. *mellonella* larvae in the different treatment groups was evaluated. **(C)** Treatment with ISL at doses of 20 or 40 mg/kg significantly improved survival rates to 60% and 70%, respectively. **(D, E)** Bacterial colony counts from homogenized larvae at 24 hours posttreatment revealed a 50% reduction in the bacterial load in the ISL-treated groups compared with the untreated control. ***, P < 0.001.

To assess the therapeutic potential of ISL, we utilized the *Galleria mellonella* model, a widely recognized *in vivo* system for evaluating antimicrobial efficacy due to its immune system’s remarkable similarity to that of vertebrates. The impact of ISL on *S. aureus* USA300 infection was evaluated by monitoring larval activity, pupation, melanization, and survival over a five-day period following infection and treatment. As shown in **Figure 5A**, survival and the bacterial load were assessed after 120 hours of infection and ISL treatment. In the wild-type (WT) group, the larvae succumbed to the infection within 120 hours, indicating pronounced melanization (**Figure 5B**). In contrast, the SrtA mutant group presented a mortality rate of only 20% at the same time point. Treatment with ISL at doses of 20 or 40 mg/kg significantly improved survival rates, which reached 60% and 70%, respectively (**Figure 5C**). To further investigate the effect of ISL on the bacterial load, larvae from each treatment group were sterilized and homogenized, and bacterial colony counts were performed 24 h posttreatment. As depicted in **Figures 5D, E**, ISL treatment led to a marked reduction in bacterial load, with a 40% decrease in *S. aureus* colony counts compared with those of the untreated control group. These findings indicate that ISL not only enhances the survival of *G. mellonella* larvae following *S. aureus* infection but also attenuates melanization.

### 3.6 Effect of ISL on mortality in mice with *S. aureus*-induced pneumonia

**Figure 6:**
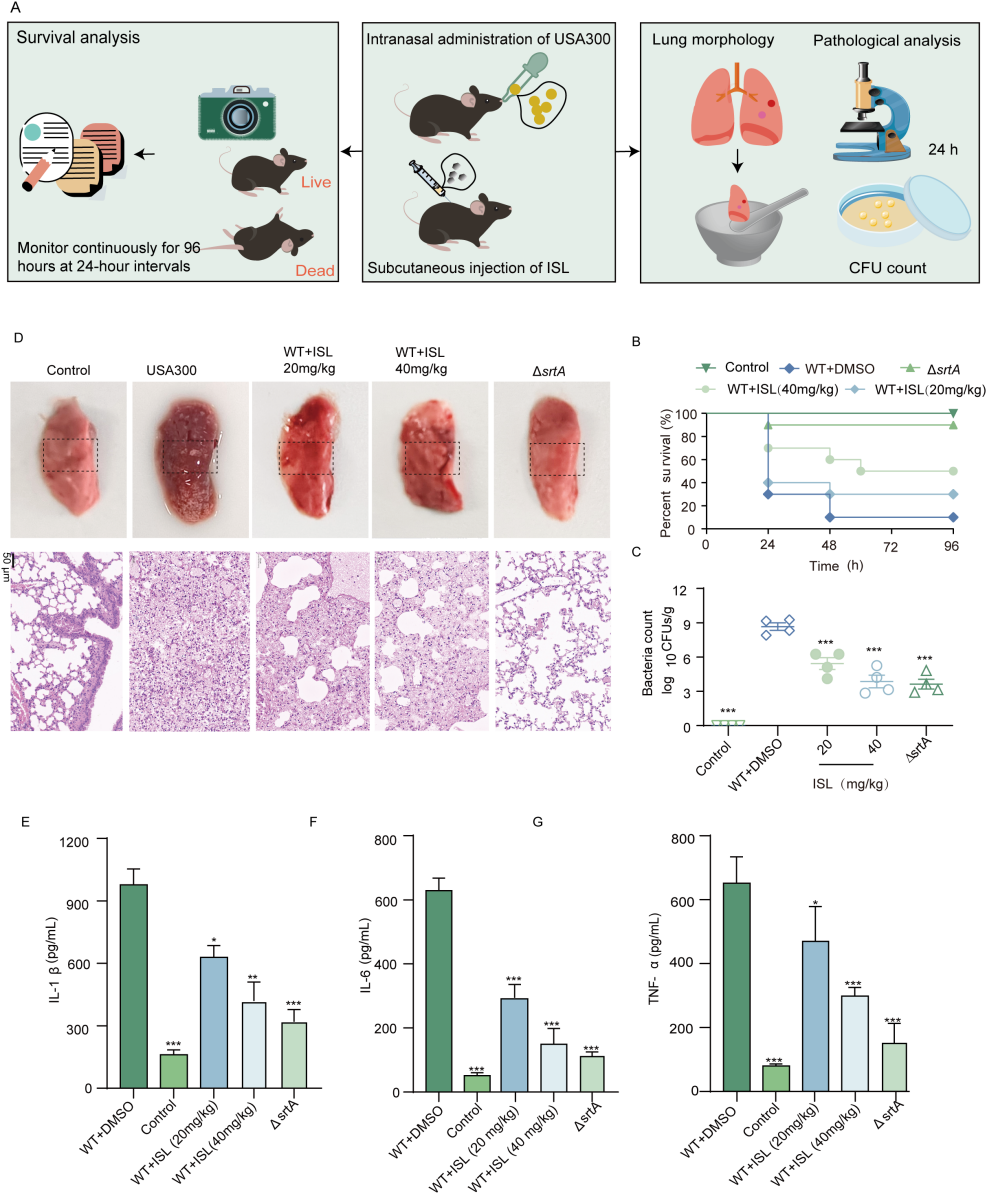
Effect of ISL on mortality in mice with *S. aureus*-induced pneumonia. **(A)** Mice were intranasally inoculated with a lethal dose of *S. aureus* USA300 and treated with 20 or 40 mg/kg ISL starting 2 h post infection. Survival was monitored over 96 h, or lung tissue was collected after 24 h for bacterial colony counts. **(B)** Survival analysis revealed that, compared with the control treatment (saline-treated) and the ΔsrtA strain (10% mortality), ISL treatment significantly improved survival, with 50% survival at 96 h. **(C)** Lung tissue bacterial load was assessed 24 h post infection. Compared with infection alone, ISL treatment reduced bacterial colonization. **(D)** Histopathological examination revealed amelioration of lung damage in ISL-treated mice, with reduced redness, swelling, and darkening compared with those in the infection group. **(E-G)** ISL treatment significantly reduced the levels of proinflammatory cytokines (IL-1β, IL-6, and TNF-α) in bronchoalveolar lavage fluid (BALF). *, P < 0.05; **, P < 0.01; ***, P < 0.001.

To investigate the *in vivo* efficacy of ISL against MRSA, we first established a murine pneumonia model induced by *S. aureus* USA300. The impact of ISL treatment on the mortality rate of infected mice was then assessed. Seven-week-old mice were intranasally inoculated with a lethal dose of *S. aureus* USA300 (2 × 10^8^ CFU). Two hours post infection, the mice were treated with 40 mg/kg ISL, followed by repeated dosing every 12 h. The survival rate was monitored over a 96-hour period. An additional group was infected with 1 × 10^8^ CFU of *S. aureus* USA300 to evaluate the extent of lung damage posttreatment (**Figure 6A**). Survival analysis revealed that Δ*srtA* presented a mortality rate of 10% within 96 hours. The control group (saline-treated) showed no mortality over the same period. In contrast, ISL-treated mice presented a significantly improved survival rate, with 50% survival by 96 h. These findings suggest that ISL enhances the survival of mice during acute pulmonary infection, particularly in the early stages of infection (**Figure 6B**). The morphology and bacterial load of the lung tissue were also evaluated. Forty-eight hours postinfection, the lung tissue was homogenized and plated, and the colony counts were determined. Compared with the infection group, the Δ*srtA* group presented minimal bacterial adhesion, with a significantly lower bacterial load (3.63 ± 0.85 log10 CFU/g) and a high bacterial burden (8.66 ± 086 log10 CFU/g). ISL treatment reduced the bacterial load in the lung tissue to 3.86 ± 1.10 log10 CFU/g, demonstrating its ability to decrease bacterial colonization in the lungs and suggesting its therapeutic potential against *S. aureus* pneumonia **Figure 6C**. Furthermore, ISL treatment ameliorated the pathological changes in the lung tissue, including redness, swelling, and darkening, which were observed in the infection group, whereas the Δ*srtA* group presented significant histopathological improvement. These findings further underscore the critical role of SrtA in the pathogenesis of *S. aureus* pneumonia (**Figure 6D**). Additionally, we assessed the levels of proinflammatory cytokines in the BALF. Treatment with ISL after *S. aureus* USA300 infection significantly reduced the levels of IL-1β, IL-6, and TNF-α (**Figures 6E-G**). Taken together, these results demonstrate that ISL exerts a potent therapeutic effect on *S. aureus* pneumonia by modulating both the bacterial load and the host inflammatory response.

## 4 Discussion

The emergence of multidrug-resistant pathogens, particularly MRSA, has raised significant global health concerns because of their high virulence and associated mortality rates. The World Health Organization (WHO) has classified MRSA as a high-priority pathogen for which novel therapeutic strategies are urgently needed (Lake et al., 2018). *S. aureus*, a versatile pathogen, is capable of inducing a wide range of infections, ranging from superficial skin lesions to life-threatening systemic diseases, including endocarditis, osteomyelitis, and sepsis (Liu et al., 2019; Wu et al., 2019). While antibiotics have long been the cornerstone of *S. aureus* infection treatment, the overuse and misuse of these agents have contributed to the widespread emergence of resistant strains, rendering many first-line antibiotics ineffective (Galar et al., 2019). This has escalated the clinical challenge of treating MRSA infections, emphasizing the need for innovative therapeutic approaches, including antivirulence strategies (Nayak et al., 2013, 2018).

The increasing resistance of MRSA to traditional antibiotics necessitates the exploration of alternative approaches to combat these infections. Antibiotic adjuvants, bacteriophage therapy, antimicrobial peptides, and nanoparticles have all been proposed as potential alternatives, but the development of antivirulence agents that specifically target bacterial pathogenicity mechanisms holds particular promise. By disarming the pathogen rather than killing it outright, antivirulence therapies aim to mitigate bacterial infection while reducing the selective pressure for resistance development (Vandenesch et al., 2012). This approach is particularly relevant for MRSA, whose virulence is largely attributed to its ability to express a diverse array of virulence factors, including surface proteins that mediate adhesion, invasion, and biofilm formation. Targeting these virulence factors provides a pathway for treating infections without compromising the entire microbiome or promoting resistance.

In particular, surface proteins play crucial roles in enabling *S. aureus* to adhere to host cells, invade tissues, and evade the immune response. These surface proteins, including fibronectin-binding proteins (FnBPA and FnBPB), clumping factors (ClfA and ClfB), and staphylococcal protein A (SpA), are key to the ability of *S. aureus* to establish and maintain infection (Foster, 2019; Ganesh et al., 2011; Stewart and Costerton, 2001; Yan and Bassler, 2019). The adhesion of bacterial products to host tissues is one of the most critical steps in initiating colonization and infection. *S. aureus* surface proteins typically recognize and bind to components of the extracellular matrix (ECM), such as fibronectin, fibrin, and collagen, facilitating bacterial adhesion and immune evasion. Microbial surface components recognizing adhesive matrix molecules (MSCRAMMs) are anchored on the cell wall peptidoglycan (PGN), and their covalent anchoring is mediated by *S. aureus* sortase enzymes, which cleave the carboxyl-terminal LPXTG motif of these surface proteins (Hati et al., 2015). Notably, ClfA binds to soluble fibrinogen and inhibits complement-mediated phagocytosis, further enhancing the pathogenic potential of *S. aureus*. Apart from SrtA, the SrtB enzyme constitutes another strategic node in the virulence network of S. aureus. Functioning as a membrane-associated transpeptidase, SrtB selectively processes proteins bearing NPQTN motifs, with IsdC being its principal substrate (Mazmanian et al., 2002). IsdC serves as a critical relay in the Isd system, enabling efficient heme capture and iron trafficking under iron-restricted host conditions (Wang et al., 2018). Perturbation of SrtB activity, either genetically or pharmacologically, impedes this process, thereby weakening bacterial iron acquisition and diminishing pathogenic fitness.

Our study demonstrated that ISL effectively targets and inhibits *S. aureus* SrtA, a crucial enzyme responsible for anchoring these surface proteins to the bacterial cell wall. By inhibiting SrtA, ISL disrupts multiple virulence mechanisms, including bacterial adhesion to host tissues, invasion of epithelial cells, and biofilm formation. FRET-based assays confirmed that ISL inhibits SrtA in a dose-dependent manner, with an IC_50_ of 13.34 μg/mL. Furthermore, kinetic studies revealed that the ability of ISL to inhibit SrtA is reversible and noncovalent, which suggests that ISL can modulate bacterial virulence without permanently altering the enzyme’s active site. This property is highly desirable for drug design, as reversible inhibition minimizes the risk of unwanted side effects, which are often associated with covalent inhibitors that irreversibly modify the target enzyme. Recent computational studies have identified several promising small-molecule inhibitors targeting alternative *S. aureus* proteins, including FmtA, the lipophilic membrane (LLM) protein, and YsxC, through structure-based virtual screening and molecular dynamics simulations (Dalal et al., 2021a; Kumari and Dalal, 2022). These targets mainly interfere with cell wall metabolism or essential protein functions, whereas ISL inhibits SrtA-mediated surface anchoring. Placing ISL within this broader context highlights its complementary mechanism and potential as a lead compound in antivirulence therapy.

The antivirulence potential of ISL was further validated through its ability to reduce the adhesion of *S. aureus* to fibrinogen and fibronectin, key components of the ECM, as well as its effectiveness in inhibiting biofilm formation. At concentrations as low as 32 μg/mL, ISL inhibited biofilm formation by both the *S. aureus* USA300 strain and the Newman strain by more than 80%. Biofilm formation is a major factor contributing to the persistence and chronicity of *S. aureus* infections, particularly in the context of medical device-related infections (Theis et al., 2023; Uberoi et al., 2024; Guo et al., 2022). The observation that ISL disrupts early biofilm formation but has limited effects on mature biofilms is clinically relevant. Early bacterial adhesion and biofilm establishment are critical initiating events in device-associated and chronic infections, such as catheter- or prosthesis-related infections, where once mature biofilms form, eradication becomes extremely difficult with conventional antibiotics or host immunity (Flemming et al., 2016; Lebeaux et al., 2013; Bjarnsholt, 2013). Therefore, the ability of ISL to interfere with the early stages of biofilm development suggests its potential utility as a preventive or adjunctive agent, particularly in peri-implant or early infection settings, to hinder biofilm establishment and reduce the risk of persistent infections.

In addition to these *in vitro* observations, the *in vivo* efficacy of ISL has been evaluated in several preclinical models. One key model employed was *Galleria mellonella* larvae, a widely used insect model that mimics the innate immune responses of vertebrates and serves as a robust platform for screening novel therapeutics (Tsai et al., 2016; Pereira et al., 2020; Champion et al., 2016; Cutuli et al., 2019). Our findings demonstrated that ISL significantly improved the survival rate of *G. mellonella* larvae infected with *S. aureus*, further confirming its antivirulence effects *in vivo*. Importantly, ISL did not have any adverse effects on larval development or melanization, two common signs of toxicity, even at doses up to 40 mg/kg. These results underscore the favorable safety profile of this compound and its potential as a nontoxic, effective treatment for *S. aureus* infections.

Most importantly, our study also evaluated the therapeutic potential of ISL in a mammalian pneumonia model, which closely resembles human disease. Pneumonia caused by *S. aureus* remains a major health concern, especially with the increasing prevalence of MRSA. In this model, the lungs of mice were infected with *S. aureus*, and the effects of ISL treatment on bacterial burden, survival rates, and histopathological changes were assessed. Compared with no treatment, treatment with ISL significantly reduced the bacterial load in the lungs, with a marked improvement in survival rates. These findings are particularly noteworthy because they suggest that ISL can effectively target *S. aureus* virulence even in a complex mammalian infection model, providing additional evidence of its therapeutic potential. Moreover, histopathological analysis of lung tissues revealed a reduction in inflammation and tissue damage, further supporting the notion that ISL acts as an antivirulence agent rather than as a bactericidal agent, allowing the host immune system to clear the infection while minimizing tissue injury. While the data from the pneumonia model are compelling, it is essential to acknowledge that this model is still in its early stages, and additional studies are needed to fully evaluate the therapeutic efficacy of ISL in more complex infection scenarios. The growing prevalence of MRSA and the diminishing efficacy of traditional antibiotics highlight the urgent need for novel strategies to combat *S. aureus* infections. In addition to its antivirulence activity, ISL may also exert direct immunomodulatory effects on the host. As a flavonoid, ISL has been reported to modulate key inflammatory signaling pathways, including NF-κB and MAPK, thereby suppressing pro-inflammatory cytokine production and oxidative stress responses (Liu et al., 2017; Li et al., 2022). This dual action—attenuating bacterial virulence while directly regulating host inflammation—may enhance its therapeutic potential against MRSA infections.

Developing effective *S. aureus* SrtA inhibitors remains challenging. The shallow and rigid active site limits pocket adaptability, and many synthetic inhibitors, such as substrate mimetics and small electrophilic compounds, show potent enzymatic inhibition but poor cell permeability and *in vivo* stability, leading to limited translational success (Shulga and Kudryavtsev, 2024; Hintzen et al., 2024). To date, no SrtA inhibitor has advanced clinically. These limitations highlight the value of natural products as alternative scaffolds with favorable bioactivity and structural diversity for inhibitor discovery (Guan et al., 2022).

Antivirulence therapies such as ISL offer a promising alternative to conventional antibiotics by specifically targeting bacterial virulence factors without exerting the selective pressure that drives resistance. This approach has the potential to complement existing antibiotics and could form the basis for a more sustainable and effective treatment paradigm. The reversible inhibition of SrtA by ISL, combined with its ability to interfere with key virulence processes such as adhesion, invasion, and biofilm formation, positions it as a strong candidate for further development as an antivirulence therapeutic. Moreover, ISL may also serve as an adjuvant to conventional antibiotics, where combining antivirulence and antibiotic strategies could enhance antimicrobial efficacy, lower required doses, and help delay the emergence of resistance. This dual potential underscores ISL’s value as both a stand-alone antivirulence agent and a supportive component of combination therapy against multidrug-resistant *S. aureus*.

## 5 Conclusion

The results presented here highlight the potential of ISL as a reversible, noncovalent inhibitor of *S. aureus* SrtA, suggesting a novel approach for the treatment of *S. aureus* infections. By targeting bacterial virulence rather than bacterial survival, ISL minimizes the risk of resistance development while effectively interfering with critical processes such as adhesion, invasion, and biofilm formation. The favorable safety profile of ISL, coupled with its strong antivirulence effects both *in vitro* and *in vivo*, suggests that it could serve as a valuable component of future therapies aimed at mitigating the threat of MRSA and other pathogenic *S. aureus* strains. Additionally, its efficacy in a mammalian pneumonia model further emphasizes its potential for clinical application, providing a compelling case for continued development and investigation as part of a broader antivirulence therapeutic strategy.

## Data Availability

The original contributions presented in the study are included in the article/**Supplementary Material**. Further inquiries can be directed to the corresponding authors.
