# Supplementary material for: Isoliquiritigenin Attenuates Staphylococcus aureus Adhesion and Invasion to Counteract Staphylococcus aureus Pathogenicity and Infection
Source: Front Cell Infect Microbiol. 2025 Nov 14;15:1686699. doi: 10.3389/fcimb.2025.1686699 (PMC12660299; doi:10.3389/fcimb.2025.1686699)
Supplement: Supplementary file 1 [file Table1.docx]

Supplementary Materials

Isoliquiritigenin Attenuates *Staphylococcus aureus* Adhesion and Invasion Mechanisms to Counteract *Staphylococcus aureus* Pathogenicity and Infection

Lili Tian^1,2#^, Jian Sun^3,#^, Hong Jiang^1,2^, Hongjun Wang^1,2^, JianZheng^1,2^, Dacheng Wang^4,^ *, Libo Zhang^1,2^*

^1^ College of Animal Science and Veterinary Medicine (Affiliated Animal Hospital), Jinzhou Medical University, Jinzhou, Liaoning, China.

^2^ Liaoning Provincial Key Product Quality and Laboratory of Animal Safety, Jinzhou, Liaoning, China.

^3^Department of Animal Husbandry and Veterinary Medicine, Beijing Vocational College Agriculture.

^4^ College of Animal Science, Jilin University, Changchun, China.

Supplementary Figure 1. Minimal inhibitory concentration of ISL against *S. aureus* Newman.

Supplementary Figure 2. Solvent-accessible surface area (SASA) analysis of the SrtA protein during molecular dynamics simulation.

Supplementary Figure 3. Time-resolved secondary structure analysis of the SrtA protein during a 100 ns molecular dynamics simulation

Supplementary Figure 1. Minimal inhibitory concentration of ISL against *S. aureus* Newman.


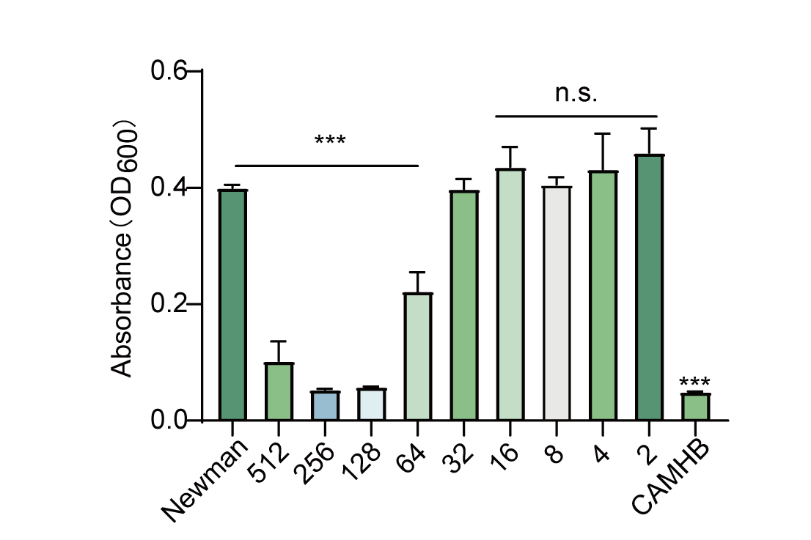


Supplementary Figure 1. Minimal inhibitory concentration of ISL against *Staphylococcus aureus* Newman. The MIC of ISL against *S. aureus* Newman under the present conditions was 128 µg/mL.

Supplementary Figure 2. Solvent-accessible surface area (SASA) analysis of the SrtA protein during molecular dynamics simulation.


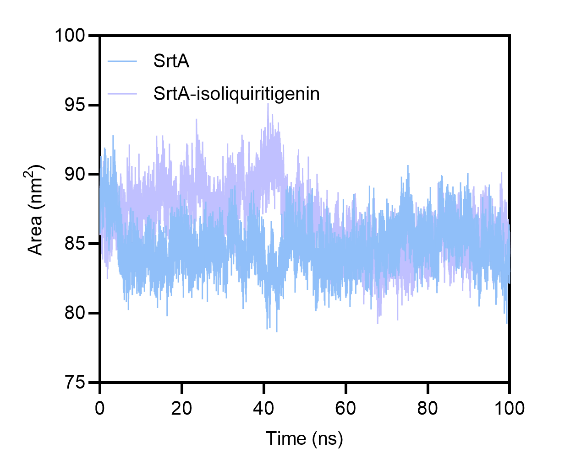


Supplementary Figure 2. Solvent-accessible surface area (SASA) analysis of the SrtA protein during molecular dynamics simulation. SASA profiles over a 100 ns simulation showing the dynamic changes in the solvent exposure of the SrtA protein in the isoliensinine-bound state (purple) compared with the apo form (blue). The observed reduction in SASA upon isoliensinine binding suggests a more compact protein conformation and potential stabilization of the SrtA structure by the ligand.

Supplementary Figure 3. Time-resolved secondary structure analysis of the SrtA protein during a 100 ns molecular dynamics simulation


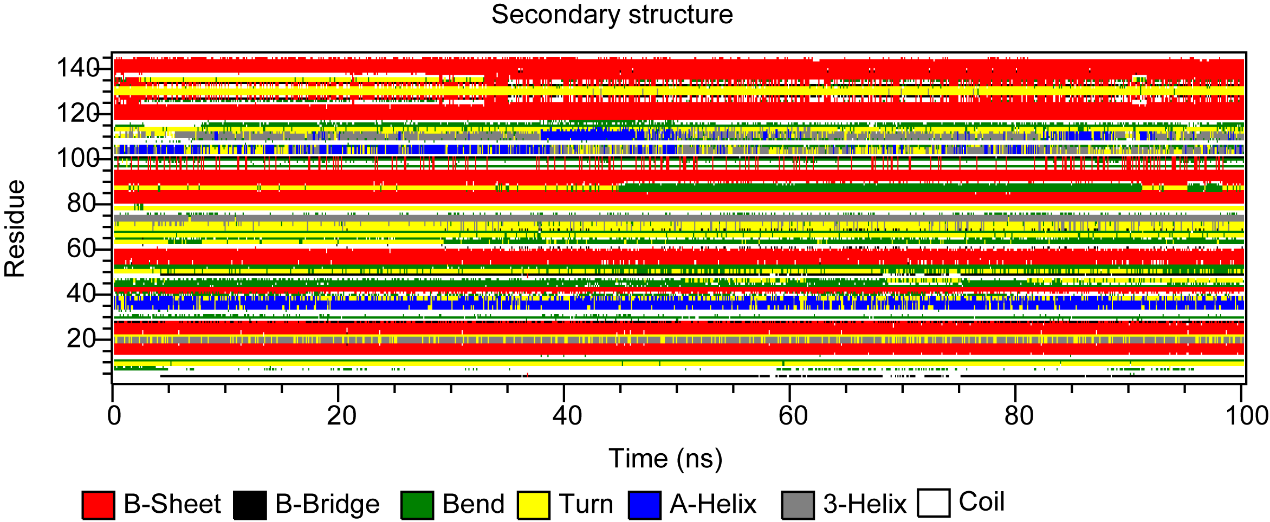


Supplementary Figure 3. Time-resolved secondary structure analysis of the SrtA protein during a 100 ns molecular dynamics simulation. The structural evolution of individual residues was monitored to assess the stability and transitions of secondary elements over time. The protein predominantly maintained β-sheet (red) and α-helix (blue) conformations, with minor fluctuations in the turn (yellow), bend (green), and coil (white) regions. The consistent secondary structure patterns suggest the conformational stability of SrtA throughout the simulation trajectory.
